# Beyond energy balance regulation: The underestimated role of adipose tissues in host defense against pathogens

**DOI:** 10.3389/fimmu.2023.1083191

**Published:** 2023-03-02

**Authors:** Johanna Barthelemy, Gemma Bogard, Isabelle Wolowczuk

**Affiliations:** Univ. Lille, Centre National de la Recherche Scientifique (CNRS), Institut National de la Santé et de la Recherche Médicale (Inserm), Centre Hospitalier Universitaire de Lille (CHU Lille), Institut Pasteur de Lille, U1019 - UMR 9017 - Center for Infection and Immunity of Lille (CIIL), Lille, France

**Keywords:** adipose tissue, immune cells, pathogens, influenza, SARS-CoV-2

## Abstract

Although the adipose tissue (AT) is a central metabolic organ in the regulation of whole-body energy homeostasis, it is also an important endocrine and immunological organ. As an endocrine organ, AT secretes a variety of bioactive peptides known as adipokines – some of which have inflammatory and immunoregulatory properties. As an immunological organ, AT contains a broad spectrum of innate and adaptive immune cells that have mostly been studied in the context of obesity. However, overwhelming evidence supports the notion that AT is a genuine immunological effector site, which contains all cell subsets required to induce and generate specific and effective immune responses against pathogens. Indeed, AT was reported to be an immune reservoir in the host’s response to infection, and a site of parasitic, bacterial and viral infections. In addition, besides AT’s immune cells, preadipocytes and adipocytes were shown to express innate immune receptors, and adipocytes were reported as antigen-presenting cells to regulate T-cell-mediated adaptive immunity. Here we review the current knowledge on the role of AT and AT’s immune system in host defense against pathogens. First, we will summarize the main characteristics of AT: type, distribution, function, and extraordinary plasticity. Second, we will describe the intimate contact AT has with lymph nodes and vessels, and AT immune cell composition. Finally, we will present a comprehensive and up-to-date overview of the current research on the contribution of AT to host defense against pathogens, including the respiratory viruses influenza and SARS-CoV-2.

## Introduction

Adipose tissues (ATs) are organized to form one of the largest organ in the body that contributes to several essential functions of our organism including e.g., mechanical support, thermoregulation, energy storage and release, regulation of appetite, and modulation of immunity (1–4). There are three major types of adipose tissues – the white adipose tissue (WAT), the brown adipose tissue (BAT), and the beige adipose tissue. WAT is the most prevalent of the tissue types and its primary role is to control the storage and release of energy to supply the bioenergetic needs to other organs. BAT is the major site of sympathetically activated nonshivering thermogenesis during cold exposure and after spontaneous hyperphagia, thereby controlling whole-body energy expenditure and body fat (5). Remarkably, WAT has the ability to reversibly acquire thermogenic characteristics in response to certain stimuli in a process called “browning” or “beiging”, corresponding to the beige (or brite) adipose tissue (6).

Since 20 years, it has been shown that WAT contains nearly every innate and adaptive immune cell types (7–10). Thus, WAT can now be considered as a true immunological organ, with important roles in anti-microbial defense, wound healing, and inflammation (11). Akin to WAT, immune cells have been recently reported to also infiltrate and reside within thermogenic ATs (i.e., BAT and beige adipose tissue) where they contribute to tissue’s homeostasis and activation (12–14). Besides, not only do ATs contain immune cells, but even adipocytes (ATs’ main constituent cells) can produce factors with immunoregulatory and antimicrobial activities (15–18), and can act as antigen-presenting cells to initiate adaptive immune responses (19).

Here, after a description of the key features of ATs: their type, distribution, function and remarkable flexibility, we review the key literature illustrating the immune properties of ATs and describe the important, yet underestimated, role ATs may play in the host defense against pathogens, notably in influenza and SARS-CoV-2 respiratory infections.

## Key features of adipose tissues: Type, distribution, function and plasticity

ATs are specialized connective tissues that are organized to form a large organ with discrete anatomy, specific vascular and nerve supplies, complex cytology, and high physiological plasticity (1, 20, 21). ATs contribute to many of an organism’s basic needs, including energy metabolism, thermogenesis, and protection against pathogens (1, 2, 4, 22).

Below, we will evoke the main characteristics of ATs: their type, location, function and exceptional capacity to adapt to various nutritional, hormonal and environmental changes; these have been extensively reviewed elsewhere (21, 23–28).

### The different types, locations, and functions of adipose tissues

In mammals, two broad categories of ATs i.e., WAT and BAT, function antagonistically to control systemic energy homeostasis (29, 30). The anabolic WAT collects, stores, and releases energy in the form of lipids (31), whereas the catabolic BAT oxidizes lipids to produce heat – a process known as adaptive thermogenesis (32–34). Functionally distinct from WAT and BAT, the bone marrow adipose tissue [also referred to as “yellow adipose tissue” or “marrow adipose tissue” (MAT)] is a key regulator of bone metabolism (35, 36). Although less well-documented than WAT and BAT, MAT has also been acknowledged as contributing to systemic energy metabolism regulation (37–40).

**Figure 1:**
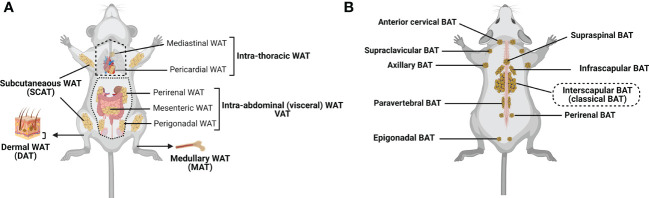
Major adipose tissue depots and anatomical locations in the mouse. There are two major types of adipose tissue: lipid-rich white adipose tissue (WAT; involved in energy storing) and mitochondria-rich brown adipose tissue (BAT; involved in energy burning) (24, 29, 30). Besides, WAT can convert to metabolically active fat through the process of browning (Beige/brite adipose tissue that is considered as the third adipose tissue type; inducible, energy expending) (6, 43). **(A)** WAT is found in many anatomical locations. The largest WAT depots are subcutaneous (SCAT; for example, inguinal, gluteal and femoral) and visceral (VAT; within the abdominal cavity, between the organs; for example, perirenal, mesenteric and perigonadal VAT). Intrathoracic adipose tissue is an extra-abdominal WAT depot located in the thoracic cavity (for example, pericardial and mediastinal WAT). Smaller WAT depots are also found around blood vessels (not shown), within the bone marrow (medullary WAT: MAT), in the dermis (dermal WAT: DAT), or as ectopic depots within specific organs (pancreas, skeletal muscle, liver (not shown)). **(B)** Classical brown adipocytes are contained in the interscapular BAT depot. Clusters of brown adipocytes are also found in other locations, including infrascapular, cervical, supraclavicular, axillary, paravertebral, epigonadal, perirenal and supraspinal depots (24, 41, 44–46). Created with BioRender.com.

WAT is the most abundant form of ATs. It can be subdivided into subcutaneous (SCAT) and visceral (VAT) adipose tissue (4, 41, 42), although smaller depots are scattered throughout the body, surrounding organs and lymph nodes (**Figure 1A**). SCAT and VAT differ significantly with regard to their cellular, molecular, and physiological characteristics, and oppositely contribute to the metabolic syndrome (2, 47, 48). Indeed, while increased amount of SCAT is associated with improved insulin sensitivity (49), VAT accumulation is commonly associated with increased insulin resistance, high-risk of type 2 diabetes, and high mortality (50, 51). SCAT is located beneath the skin, and should be distinguished from the less-characterized dermal WAT (DAT), which resides directly below the reticular dermis (i.e., above the SCAT), and is involved in thermal insulation, hair regeneration, wound healing and protection against skin infections (17, 18, 52–55). VAT includes intrathoracic fat depots (surrounding the heart: epicardial and pericardial fat, situated within the mediastinum: mediastinal fat), and intraabdominal fat depots (mesenteric, omental, perigonadal, perirenal and retroperitoneal fat). In addition, under certain physiological (e.g., aging) or pathological (e.g., metabolic syndrome) conditions, fat depots can also develop around or within skeletal muscles (56) and in liver (57, 58).

Compared to WAT, BAT is located in more specific areas (41, 44, 45). In rodents, the largest and most investigated BAT depot is the subcutaneous interscapular BAT, but BAT depots can also be found in the supraclavicular region of the neck (46), and in perirenal region (45) (**Figure 1B**). In humans, the supraclavicular region contains the highest proportion of total body BAT volume, followed by the mediastinal, thoracic paravertebral, perinephric, and adrenal loci (59). While previously believed to be nonexistent or nonfunctional in adult humans, several reports provide unequivocal evidence of the presence and activity of BAT in this population (60, 61).

In stark contrast to WAT and BAT, MAT has a specific location in the bone marrow of certain long bones and vertebrae, where it resides side-by-side with hematopoietic and bone cells (36, 40, 62, 63). MAT includes two distinct subtypes that have different locations in bones – the “constitutive MAT”, concentrated in the distal skeletal bones, and the “regulated MAT” that is diffusely distributed in the spine and proximal limb bones, and is regulated by several environmental factors (40, 64).

### The remarkable plasticity and remodeling capacity of adipose tissues: Introducing the beige adipose tissue

A momentous property of ATs is their high degree of plasticity. To meet the organism’s needs, ATs’ size, metabolism, structure, and phenotype can change rapidly (25–27, 65).

The potential for ATs to grow and regress in size is substantial. WAT can quickly expand its volume within days after initiation of an obesogenic diet through an increase in the size (hypertrophy, due to enhanced lipid storage capacity) and/or the number (hyperplasia) of adipocytes, whereas it rapidly shrinks by lipolysis upon fasting or cold exposure (66–69). This illustrates (*i*) the metabolic plasticity of WAT i.e., the ability to switch between two opposing metabolic programs: nutrient storage (lipogenesis) vs. nutrient release (lipolysis), and (ii) the cellular plasticity of WAT since newly generated adipocytes can be recruited during obesity development and relapse, as well as during cold exposure (65, 70–73).

As observed in obesity, aging is also associated with expanded WAT mass (resulting from decreased SCAT and DAT mass, and marked increased VAT mass), and reduced BAT mass and activity (74–77). At the molecular level, transcription control through forkhead box protein A3 (FOXA3) has been proposed as a potential regulatory factor for WAT accretion and BAT decline during aging and obesity (78). In addition, it has been reported that aging and obesity are also associated with increased MAT mass (79, 80). Most importantly, the age- and obesity-related changes in ATs’ mass and location are associated with progressive dysfunction of these tissues, ultimately leading to systemic inflammation and occurrence of metabolic disorders; this defines the concept of “adipaging” (77, 81, 82).

Another striking example of ATs’ remarkable metabolic plasticity is that, under various physiological, pathological and pharmacological conditions, WAT can acquire oxidative BAT-like features and, conversely, that BAT can convert into WAT-like tissue, in processes respectively termed “WAT browning” (or “beiging”) (6, 43) and “BAT whitening” (83). Thermogenic adaptation of ATs has been initially described during environmental cold exposure: BAT mass increases together with elevated expression of thermogenic genes (84). In WAT, especially in rodents, cold exposure induces the development of thermogenic beige adipocytes within the tissue (84, 85). Results from several studies further support that “inducible” brown adipocytes (aka “beige” or “brite”) can emerge within classical WAT depots in response to cold acclimatation and other stimuli such as β 3-adrenergic stimulation (28, 86). The exact mechanism is currently unclear (41, 87). Some have proposed that the browning/beiging of WAT is a result of *de novo* production of beige adipocytes (88), while other studies support that beige adipocytes derive from preexisting adipocytes (89). It is noteworthy that both aging and obesity are associated with impaired WAT browning (75, 90), and enhanced BAT whitening (91). Very few studies have focused on mechanisms involved in BAT whitening. For optimal thermogenic activity, BAT requires an extensive vascularization to ensure efficient supplies of oxygen needed to support the high energy consumption (92). It has been proposed that BAT whitening may partly rely on decreased vascularity of the tissue, leading to functional hypoxia and decreased thermogenic activity (93). Moreover, beige adipocytes can transform into energy-storing white adipocytes within days after external stimuli are withdrawn (94, 95).

The biomedical interest in beige AT – now viewed as the third type of ATs, is currently centered on the capacity of this oxidative tissue to counteract obesity *via* induction of energy expenditure, and to mitigate the vast array of obesity-associated diseases, including type 2 diabetes, heart disease, insulin resistance, hyperglycemia, dyslipidemia, hypertension, and many types of cancer (96, 97).

## Adipose tissues: A proper and legitimate component of the immune system

Immune organs represent sites of exclusive immunological function, however, the definition of an immune tissue has been extended to organs such as the liver (98), uterus (99), skeletal muscle (100), hypothalamus (101), and small intestine (102). Similarly, ATs (mostly described for WAT) are now defined as proper immune organs (3, 11, 103, 104).

### The adipose-lymphatic crosstalk

The lymphatic system is distributed throughout the body and consists of lymphoid nodes and organs, and lymphatic vessels (105, 106). The lymphatic system forms a one-direction transit pathway from the extracellular space toward the venous circulation to maintain fluid homeostasis by removing the protein-rich lymph from the extracellular space among tissues and returning it to the bloodstream (107). In addition to its fluid homeostasis function, the lymphatic system is also important for transport of pathogens [bacteria, viruses, prions (108)], antigens, exosomes and immune cells (including antigen-presenting cells) to regional lymph nodes and lymphoid structures, and management of immune cell trafficking and inflammation (107, 109–114). Besides, it has been reported that lymphatic endothelial cells regulate immune responses more directly by controlling entry of immune cells into lymphatic capillaries, presenting antigens on major histocompatibility complex proteins, and modulating antigen presenting cells (115).

In humans, lymphatic density varies greatly from AT depot to depot, but local lymphatic vessel function may still impact local adipose health (116, 117). In mice, lymphatics are absent in BAT and present in WAT (rare in gonadal VAT, sparse in SCAT). For instance, SCAT lies in proximity to the dermal lymphatic vasculature, and VAT surrounds the collecting lymphatic vessels of the mesentery, cisterna chyli and thoracic duct, as well as the efferent and afferent lymphatic vessels of intra-abdominal lymph nodes (118).

In addition, a close relationship exists between ATs and lymph nodes – the organizing centers of immune surveillance and response. Indeed, even in the leanest animals, lymph nodes are always found surrounded by WAT (119). Extensive work by Pond and colleagues further revealed the bidirectional functional partnership connecting WAT and lymph nodes: through increasing its rate of lipolysis, perinodal WAT serves as a reservoir of energy that is deployed to power local immune responses while, vice-versa, chronic lymph node activation results in increased WAT mass (119–124).

### All immune-cell-types are present in adipose tissues and functionally active

Despite a rather simple histological appearance, ATs’ cellular composition is complex (125). Although contributing to more than 90% of ATs’ volume, mature, lipid-filled adipocytes represent less than 50% of adipose cells (126). Other cell types in ATs (collectively referred to as stromal-vascular cells) include e.g., multipotent mesenchymal progenitor cells, preadipocytes, and a broad spectrum of innate and adaptive immune cells – which all spatially and functionally interact. Interest in adipose immune cells was significantly accelerated by the discovery that ATs’ immune cell composition is highly sensitive to metabolic and nutritional states (7, 8); this exemplifies another aspect of ATs’ cellular adaptability. Although mainly described for WAT, in which they contribute to the regulation of systemic metabolism by modulating the inflammatory tone of the tissue, immune cells are also present in thermogenic ATs (i.e., BAT and beige adipose tissue) where they have been proposed to support and regulate tissue remodeling and thermogenic function (12–14, 127, 128).

AT’s innate and adaptive immune cell types and functions have been extensively documented, mainly for WAT and predominantly in the context of obesity (7–10, 129). In the lean state, WAT contains anti-inflammatory M2-like macrophages, regulatory T cells (T_reg_ cells), type 2 innate lymphoid cells (ILC2s), invariant natural killer cells (iNKT cells), natural killer cells (NK cells), eosinophils, dendritic cells (DCs), and γδ T cells, which all cooperate to prevent inflammation and coordinate metabolic responses. In the obese state, the profile of adipose immune cells shifts towards a proinflammatory-type of cells: the number of neutrophils, inflammatory M1 macrophages, mast cells, B cells, DCs, CD8^+^ T cells, T helper (Th) 1 cells and Th17 cells increases, while the number of eosinophils, iNKT cells, ILC2s and T_reg_ cells decreases (9, 129). Interestingly, in the context of mouse and human obesity it has been reported that immune cell composition differ between SCAT and VAT (130–132).

A summary of immune cells identified in ATs is presented in **Table 1**.

**Table 1:** Immune cells (innate, adaptive and bridging innate & adaptive immunity) found in adipose tissues.

Type of Immunity	Immune cells	References
**Innate Immunity**	Macrophages (M1, M2) Neutrophils Mast cells Eosinophils Dendritic cells NK cells	(7, 133–142)
**Bridge between Innate & Adaptive Immunity**	iNKT ILCs (ILC1s, ILC2s) γδ T cells MAITs	(143–150)
**Adaptive Immunity**	B cells CD4^+^ T cells (Th1, Th2, Th17, T_reg_ cells) CD8^+^ T cells	(132, 151–157)

In addition, it has to be mentioned that some intraabdominal and intrathoracic VAT depots are rich in immune cell clusters called fat-associated lymphoid clusters (FALCs). FALCs correspond to inducible, atypical lymphoid tissues that were first identified in the omentum where they were termed “milky spots” (158). Later, the presence of FALCs was reported in other VAT depots (i.e., mesenteric, mediastinal, gonadal and pericardial) (159–161). FALCs are in direct contact with adipocytes, lack fibrous capsule (unlike lymph nodes), and contain B cells, T cells, macrophages, dendritic cells, NKT cells, iNKT cells and ILC2s (159). Importantly, FALCs can give rise to germinal centers under certain conditions (162). These small clusters of immune cells behave as a secondary lymphoid organ and are responsible for modulating both innate and adaptive immune responses (163). The presence of FALCs, together with the aforementioned resident adipose immune cells, have led some authors to propose that ATs can be considered as a tertiary lymphoid organ, with hallmarks of innate and adaptive immune responses (164).

The mechanisms by which ATs contribute to immune responses may be *via* direct effects of adipose immune cells, and/or *via* indirect effects whereby adipocytes modulate immunity and inflammation through endocrine, paracrine, autocrine or juxtacrine mechanisms of action. As endocrine organs, ATs communicate with other organs (including immunological organs) *via* the synthesis and secretion of a multitude (>600) of molecules typically referred to as “adipokines”, such as TNFα, IL-6, chemerin, resistin, visfatin, vaspin, irisin, omentin-1, lipocalin-2, apelin, adiponectin and leptin, to name but a few (15, 50, 165–167). In ATs, adipokines can be released by adipocytes, preadipocytes, adipose resident and -infiltrated immune cells, or other cell types. In addition to their regulation of ATs’ metabolic homeostasis, some adipokines have immunoregulatory and inflammatory functions. For example, adiponectin has anti-inflammatory properties (168) and can negatively regulate macrophage function (169). In contrast, leptin is a proinflammatory factor (170) with broad actions on both the innate (activation of monocytes/macrophages, neutrophils and NK cells) and adaptive (promotion of CD4^+^ T cell proliferation and IL-2 secretion) immunity (171, 172).

In addition, accumulating evidence indicates that adipocytes can behave as immune cells and sense inflammatory cues; thereby playing an important role in shaping immune responses. Indeed, adipocytes have the ability to express innate immune receptors (173), produce proinflammatory cytokines (50), express chemokines (174), and present antigens (175–177). Most recently, Caputa et al., reported that adipocytes can be licensed by adipose innate immune cells (i.e., NK cells and iNKT cells) to acquire anti-bacterial functions during *Listeria monocytogenes* infection; this highlights the extra-metabolic capacity of adipocytes to actively participate in the immune response to bacterial infection (178). Such fascinating findings highlight the intricacy and potential for adipocytes to robustly modulate ATs and systemic inflammation that in turn impact global immune responsiveness under homeostatic and disease state conditions.

## Adipose tissues and host defense against microbial infections

Unprecedented changes are occurring worldwide: populations age and the prevalence of obesity and related comorbidities continue to increase. This represents a major health challenge since obesity and aging both predispose to health complications (including diabetes, cardiovascular diseases, and cancer), and increase the risk of infections [including respiratory infections, such as influenza (179, 180) and COVID-19 (181)] and premature death. Both obesity and aging are characterized by enhanced low‐grade chronic inflammation and altered innate and adaptive immune cell functions, which contribute to the impaired immune surveillance and host defense in obese and aged individuals (182). Another feature is shared by obesity and aging i.e., the pathological expansion of WAT in general and VAT depot in particular, together with changes in adipose immune cell composition (48, 183); this suggests that WAT remodeling occurring in aged and obese individuals may contribute to the markedly increased vulnerability of these at-risk populations.

Next, we will show that ATs can be targeted by several pathogens that can eventually accumulate and persist in the tissue, and even be a site of active immune responses to infection.

### Adipose tissues can be “the place-to-be-and-stay” for some pathogens

Pathogens that have been reported to infect ATs, and eventually persist within, are listed in **Table 2**.

**Table 2:** Pathogens found in adipose tissues.

Species	Disease	Type of ATs	ATs’ targeted cells	Refs
***Mycobacterium tuberculosis***	Tuberculosis	- WAT (SCAT, VAT, perirenal, pericardial,perinodal, mesenteric AT) - BAT?	- Adipocytes - Stromal vascular cells (preadipocytes, CD8^+^T cells)	(184–186)
***Coxiella burnetii***	Q fever	- WAT (SCAT, VAT) - BAT	- Adipocytes - Stromal vascular cells (macrophages)	(187)
***Rickettsia prowazekii***	Epidemic typhus	- WAT - BAT?	- Adipocytes	(188)
***Listeria monocytogenes***	Listeriosis	- WAT - BAT?	- Adipocytes - Stromal vascular cells (CD8^+^T cells)	(189)
***Clamydophyla pneumoniae***	Pneumoniae	- WAT? - BAT?	- Adipocytes	(190, 191)
***Staphylococcus aureus***	Sepsis, Pneumoniae, Skin infections	- WAT - not BAT	- Adipocytes	(192, 193)
***Trypanosoma cruzi***	Chagas disease	- WAT (SCAT) - BAT	- Adipocytes	(194–196)
***Trypanosoma brucei***	African trypanosomiasis (Sleeping sickness)	- WAT (SCAT) - BAT	- Interstitial spaces between adipocytes	(197–199)
***Plasmodium falciparum***	Malaria	- WAT (SCAT) - BAT?	- Sequestration of infected red-blood-cells inside ATs’ microvasculature	(200, 201)
***Plasmodium berghei***	Malaria	- WAT (SCAT) - BAT?	- Sequestration of infected red-blood-cells inside ATs’ microvasculature	(202)
***Leishmania infantum***	Visceral leishmaniasis	- WAT (VAT) - BAT	- Adipocytes	(203, 204)
**Adenovirus (Adv36)**	Mild-flu	- WAT (VAT) - BAT?	- Adipocytes - Stromal vascular cells (preadipocytes)	(205–208)
**HIV (SIV)**	AIDS	- WAT (SCAT & VAT) - Not BAT	- Stromal vascular cells (CD4^+^ T cells, macrophages )?	(209–213)
**Lymphocytic choriomeningitis virus**	Neurological disorders	- WAT (VAT) - Not BAT	- Adipocytes - Stromal vascular cells (T cells)	(214, 215)
**Cytomegalovirus**	Athero-sclerosis, Cardio-vascular disorders, Prostate cancer	- WAT (VAT, peri-pancreatic AT) - BAT	- Adipocytes - Stromal vascular cells (preadipocytes, CD8^+^ T cells)	(216–218)
**Influenza virus**	Flu	- WAT (SCAT, epididymal, mesenteric, peri-vascular VAT) - BAT?	- Adipocytes - Stromal vascular cells (preadipocytes, hematopoietic cells)	(219–222)
**SARS-CoV-2 virus**	COVID-19	- WAT (SCAT, epididymal, mediastinal VAT) - BAT?	- Adipocytes - Stromal vascular cells (macrophages)	(223–226)

Green shading: bacteria, blue shading: parasites, yellow shading: viruses.

WAT, White adipose tissue; SCAT, Subcutaneous adipose tissue; VAT, Visceral adipose tissue; BAT, Brown adipose tissue; H(S)IV, Human (Simian) immunodeficiency virus; AIDS, Acquired immunodeficiency syndrome; SARS-CoV-2, Severe acute respiratory syndrome coronavirus 2; COVID-19, Coronavirus disease-19.

Completed from [Tanowitz et al., (227)].

#### Bacteria

*Mycobacterium tuberculosis* (Mtb), the etiological agent of tuberculosis, has the ability to persist in its host for a long time. However, aside from the lungs and regional lymph nodes (which are the main targets for Mtb), the other locations of the bacilli in latently infected patients remain incompletely identified (228). WAT may constitute, among others, an important reservoir for *M. tuberculosis*. Indeed, Mtb could be isolated from the SCAT and several VAT depots (i.e., perigonadal, perirenal, and mesenteric) of infected mice (184). In individuals with active or latent tuberculosis, Mtb was found in perinodal WAT (active tuberculosis) and in perirenal VAT, abdominal SCAT, and perinodal WAT (latent tuberculosis) (185). Furthermore, Neyrolles and colleagues showed that Mtb, after binding to scavenger receptors, can infect adipocytes *in vitro*, where it persists in a non-replicating, dormant, state (185). Importantly, the presence and persistence of Mtb in WAT was shown to modulate adipose tissue’s physiology. *M. tuberculosis* infection drives the recruitment of Mtb-specific IFNγ^+^ CD8^+^ T cells and IFNγ^+^ NK cells into the WAT, indicative of local inflammation (186). More recently, it has been reported that *M. tuberculosis* infection also stimulates the infiltration of inflammatory immune cells in BAT (229). Interestingly, infection was associated with adipocyte hypertrophy in both WAT and BAT, thereby modulating whole-body energy metabolism (229). The important contribution of WAT to the host’s pathophysiological response to *M. tuberculosis* infection was confirmed through the use of transgenic inducible “fatless” mice (230). Using this elegant approach, the authors demonstrated that the loss of fat cells during the course of Mtb infection promote the severity of pulmonary pathogenesis. Of note that *Mycobacterium canettii*, a rare representative of the *M. tuberculosis* complex, has been reported to successfully infect preadipocytes and adipocytes *in vitro (* 231). However, while WAT is undoubtedly a reservoir for Mtb, it is an unlikely sanctuary for *M. canettii*, and it is still an open question whether *M. canettii* and *M. tuberculosis* can persist in BAT.

*Coxiella burnetii* (*C. burnetti*), the agent of Q fever, is known to persist in humans and rodents (232). Bechah and colleagues identified WAT (SCAT and VAT) and BAT as tissue reservoirs for the bacterium: *C. burnetii* can persist in mouse WAT and BAT for at least four months after infection, while it was not detected in the blood, spleen, liver or lungs as early as 30 days of infection (187). Importantly, the transfer of VAT from convalescent mice to naive immunodeficient mice resulted in the infection of the recipient animals. Analysis of *C. burnetii* localization in WAT demonstrated that the bacterium targets macrophages and adipocytes. *In vitro* infection of mouse adipocytes showed that *C. burnetii* can infect and replicate within adipocytes, where it resided in late phagosomes (187). Whether ATs are reservoirs for *C. burnetii* in humans remains to be shown.

*Rickettsia prowazekii* (*R. prowazekii*) is the causative agent of epidemic typhus, also called louse-borne typhus. Until recently, epidemic typhus was considered a disease of the past, however re-emergences have been reported (233). *R. prowazekii* can infect and replicate in adipocytes *in vitro*, and the bacterium was detected in murine WAT, but not in liver, spleen, lung, or central nervous system, up to four months after recovery from the primary infection, suggesting a role for WAT as a potential reservoir for dormant infections with *R. prowazekii (* 188). Whether ATs are reservoirs for *R. prowazekii* in humans remains to be shown.

*Listeria monocytogenes* (*L. monocytogenes*) is a food-borne bacterium responsible for a disease called listeriosis, which is potentially lethal in immunocompromised individuals (234). It has been reported that *L. monocytogenes* can infect adipocytes *in vitro*, inducing the upregulation of the expression of the genes coding monocyte chemoattractant protein-1 (MCP-1, involved in macrophage recruitment) and adiponectin (an anti-inflammatory, insulin-sensitizing adipokine) (189). In addition, *L. monocytogenes* was detected in the WAT of infected obese mice (189). Recently, Caputa and colleagues explored the role of perinodal WAT in the immune response to *L. monocytogenes (* 178). Contrary to their expectations, perinodal WAT was not required for the development of the adaptive immune response during infection. However, the authors found that, over the course of infection, perinodal WAT adipocytes became infected and were rapidly cleared of bacteria. Importantly, the authors demonstrated that infected adipocytes initiated a transcriptional response to IFN-γ (produced by adipose NK and iNKT cells) and shifted away from lipid metabolism toward anti-bacterial functions (178). These major results clearly demonstrate the repurposing of adipocytes away from lipid metabolism toward fighting infection.

*Chlamydophila pneumoniae* (*C. pneumonia*), renamed from *Chlamydia pneumoniae* in 1999, causes respiratory tract infections such as pneumonia. The potential effect of *C. pneumoniae* infection on fat cells was first investigated by Shi and colleagues in 2008: *C. pneumonia* can successfully infect preadipocytes and adipocytes *in vitro*. Strikingly, infection of preadipocytes impaired their differentiation towards fully mature and insulin-sensitive adipocytes through a TNFα-mediated inflammatory mechanism (190). Later on, it was reported that *C. pneumoniae* proliferated in *in-vitro*-infected mouse adipocytes by inducing lipolysis, thereby acquiring energy for its own replication to the detriment of host’s lipid metabolism pathway (191). The same authors further reported that *C. pneumonia*e infection robustly induces fatty acid-binding protein 4 (FABP4, an intracellular lipid chaperone) secretion from adipocytes partly by stimulating the endoplasmic reticulum stress/unfolded protein response (235). However, no reports are available yet on the detection of *C. pneumonia* in ATs.

*Staphylococcus aureus* (*S. aureus*) is a commensal bacterium and opportunistic pathogen, causing potentially fatal disease. It represents the most frequently isolated human bacterial pathogen from a range of diseases including e.g., sepsis, pneumonia, and skin infections (236, 237). *S. aureus* is able to infect adipocytes *in vitro*, and to survive inside these cells (192). Adipocyte infection with *S. aureus* decreases adiponectin and resistin release whereas visfatin, monocyte chemoattractant protein-1 (MCP-1), and IL-6 secretion are increased (192). Because adipocyte viability is not affected during infection, it has been proposed that ATs (and adipocytes within) might function as hosts for *S. aureus* chronic infection. *In vivo*, Zhang and colleagues reported massive expansion of the DAT in response to a *S. aureus* subdermal infection in mice, resulting from both increased adipocyte size (hypertrophy) and number (hyperplasia) (18). Importantly, the authors showed that the local expansion of dermal fat produces the antimicrobial peptide cathelicidin [which has been described to inhibit bacterial growth, stimulate neutrophils and exert proinflammatory activities (238)], but this response appears to decline as adipocytes mature (18). The defective cathelicidin production by mature adipocytes may explain observations of elevated susceptibility to *S. aureus* infection during obesity and insulin resistance that have been reported in experimental mouse models (239, 240) and in humans (241, 242). Another possible explanation for this apparent discrepancy is that insulin resistance, leptin resistance and/or other aspects of the metabolic syndrome may perturb the infection/adipogenesis/cathelicidin pathway identified by Zhang and colleagues (18). Data on the regulation of cathelicidin are still scarce but signaling by adipose-derived hormones such as resistin could potentially influence its expression; Hochberg and colleagues indeed reported a positive correlation between circulating cathelicidin and resistin levels in obese subjects undergoing a bariatric surgery (243). Besides, cathelicidin is post-translationally cleaved to its active form (244), however, whether this process could also be influenced by factors that are modulated in obesity and metabolic syndrome remains to be seen. In contrast to WAT, *S. aureus* infection has no impact on BAT (245). However, the relevance of these observations during *S. aureus* infection in humans still needs to be elucidated and has to be investigated in future studies.

#### Parasites

Three parasite species are uniquely associated with ATs during their life cycle: *Trypanosoma cruzi*, the causative agent of Chagas disease; *Trypanosoma brucei*, the causative agent of African sleeping sickness; and *Plasmodium* spp., the causative agents of malaria. Recently, it has been reported that *Leishmania infantum*, responsible of visceral leishmaniasis, can also reside in ATs.

In 1970, Shoemaker and colleagues first reported the presence of *Trypanosoma cruzi* (*T. cruzi*) in the BAT of infected mice (194). Later on, a high number of parasites was also reported in the WAT of infected mice (195, 196). Using acute and chronic mouse models of *T. cruzi* infection, it was subsequently demonstrated that the parasite directly infects ATs (both WAT and BAT) where it can persist, notably in adipocytes (246, 247). In humans, *T. cruzi* was found in the SCAT of patients with chronic Chagas disease, confirming the role of ATs as tissue reservoirs for the parasite from which recrudescence may occur during immunosuppression (248). The presence of *T. cruzi* in ATs is associated with local inflammation (notably increased production of IL-6 and TNFα), macrophage recruitment and oxidative stress, as well as with a reduction in lipid accumulation, adipocyte size, and fat mass partly resulting from increased expression of lipolytic enzymes (247, 249, 250).

African trypanosomiasis (also known as “sleeping sickness”), caused by *Trypanosoma brucei* (*T. brucei*), is transmitted by tsetse flies. In mammalian hosts, trypanosomes are thought to exist in two major niches: early in infection, they populate the blood and later, they breach the blood-brain barrier. Trindade and colleagues uncover ATs (both WAT and BAT) as the third, and major, niches where parasites can accumulate and replicate through their functional adaptation to lipid-rich environment (197). Since skin is the entry site for the parasite, many parasites can be found in the vicinity of SCAT adipocytes, but not in adipocytes (198, 199). Thus, like for *T. cruzi*, there is no doubt that ATs are major *T. brucei* reservoirs. However, while *T. cruzi* resides inside adipocytes, *T. brucei* is found in the interstitial spaces between adipocytes.

Five species of genus *Plasmodium* are known to cause malaria in humans: *Plasmodium falciparum*, *P. vivax*, *P. malariae*, *P. ovale*, and *P. knowlesi*. However, infection with *P. falciparum* is being accounted for more than 90% of the world’s malaria mortality (251). In addition, *P. berghei* causes malaria in certain rodents, such as mice (252 **)**. Examination of post-mortem tissues obtained from individuals who died from severe malaria revealed that *P. falciparum* accumulate in WAT (mainly SCAT), although the lung and the spleen are the main sites of parasite accumulation (200, 201). In WAT, the sequestration of *P. berghei*-infected-red-blood-cells (iRBCs) largely depends on CD36 (202) – a scavenger receptor that regulates the process of lipid storage and lipolysis (253). Importantly, it has been recently reported that iRBCs sequestration in WAT microvasculature increases the production of leptin (254), the circulating levels of which are associated with severe (i.e., cerebral) malaria in mice (255, 256). Thus far, there is no report of *Plasmodium* spp.-iRBCs sequestration in BAT.

*Leishmania* spp. are the causative agents of a spectrum of clinical diseases, all termed leishmaniasis. Two species of *Leishmania* are known to give rise to the visceral form of the disease, which is the most severe form of leishmaniasis: *L. donovani* and *L. infantum*. The report of several cases of relapses, even post-treatment, has raised the unresolved question of host sites allowing parasite persistence (257). *L. infantum* persistence in the VAT of intra-peritoneally infected mice was described in 2011 (203). Recently, it has been reported that *L. infantum* can reside and persist in the VAT and BAT of intravenously infected mice, mostly in adipocytes (204). However, it remains to be determined whether ATs and adipocytes could be a reservoir for *L. infantum* in humans.

#### Viruses

Adenoviruses (Advs) are common viruses that typically cause mild cold- or flu-like illness. More than 80 different Adv types can infect humans (258). The possibility for Advs to be associated with WAT in humans was initially considered in 1997 by Dhurandhar and colleagues, who conducted a pioneer study linking a virus to obesity in humans (259). Adv36 was the first human virus to be identified as causing obesity in animals (260), and the only virus that was related to obesity and/or metabolic alterations in naturally infected humans (261). In non-human primates, it was reported that Adv36 reside in VAT (205), an observation that has been repeatedly extended to humans (206–208). *In vitro*, Adv36 infection promotes preadipocyte differentiation toward mature, lipid-filled adipocytes (262, 263), partly through the regulation of adipogenesis-related genes such as CCAAT/enhancer-binding protein (C/EBP) α and β, peroxisome proliferator-activated γ (PPARγ) and glycerol-3-phosphate dehydrogenase (GPDH) (264). Importantly, Adv36 infection leads to the decreased production of the proinflammatory, immunoregulatory adipokine leptin (263), and the increased production of the anti-inflammatory adipokine adiponectin (265). To our knowledge, there is to date no information regarding the presence of Adv36 in BAT.

Human immunodeficient virus (HIV) is responsible for the progressive immune dysfunction that leads to acquired immunodeficiency syndrome (AIDS). The notion that ATs act as reservoirs for HIV was first put forward by Dupin and colleagues in 2002 (266). However, the identification of ATs (exclusively WAT, both SCAT and VAT) as long-lived sanctuaries for replicative viruses and major sites of inflammation during chronic HIV (and its simian homologue SIV) infection, came later (209–213). Although adipocytes express the CD4, CCR5, and CXCR4 cell surface receptors necessary for HIV entry, viruses seldomly infected cultured adipocytes and, when entry does occur, the production of viral particles is relatively poor (267, 268); adipocytes are thus not likely to be the major cell hosts in WAT. In fact, HIV (and SIV) was detected in WAT’s stromal vascular fraction (209), notably in CD4^+^ T cells (210, 212). Of note that unambiguous data on adipose macrophages are missing: the virus was found in these cells in the SIV infection model but not in samples from HIV-infected patients (212).

Cytomegaloviru*s* (CMV) is a wide-spread virus, with manifestations ranging from asymptomatic to severe end-organ dysfunction in immunocompromised patients with congenital CMV disease (269). During the last decades, CMV was implicated in the pathogenesis of atherosclerosis and cardiovascular disorders (270) as well as prostate cancer (271). CMV can infect preadipocytes and adipocytes *in vitro (* 272, 273), and it has been recently reported that mouse CMV infects VAT, resulting in the durable enrichment of cytotoxic CMV-specific CD8^+^ T cells and that carry markers of tissue residence (216). Of note that the presence of CMV in BAT has been reported in an acute model of infection in rodents (217, 218).

Lymphocytic choriomeningitis virus (LCMV) is an important cause of neurologic diseases in humans (274). It has been recently reported that LCMV infects WAT (only reported for the perigonadal VAT depot) and adipocytes (214). Virus-specific T cells accumulate in the WAT and are likely responsible for clearing infection there. These T cells then differentiate into memory T cells that appear transcriptionally distinct from memory T cells in circulation or in lymphoid tissues (214). Whether SCAT and/or BAT can also be targeted by LCMV remains unknown.

### Evidences for the contribution of adipose tissues to the host’s memory/recall response to pathogens

Tissue-resident memory CD8^+^ T cells (T_RM_) are a distinct memory population that is generated and persists at the infection site (275–277). Upon exposure to the same (or similar) pathogen, T_RM_ cells provide a first line of adaptive cellular defense and are crucial in lethal challenge models (278). In mice, non-human primates and humans, it has been shown that ATs (only reported for WAT) harbor many T_RM_ cells (279). Interestingly, WAT’s T_RM_ cells metabolically adapt to the tissue through up-regulating genes involved in lipid metabolism, indicating that they use fatty acid metabolism for their homeostasis (280).

So far, only few reports have underscored that ATs act as reservoirs for CD8^+^ T_RM_ cells: after infection (VAT) (193, 214), dietary restriction (MAT) (281), and obesity (VAT) (214, 282, 283), thereby likely participating to adaptive immune responses.

Han and colleagues reported that, following *T. gondii* or *Y. pseudotuberculosis* infection, nearly half of all CD8^+^ T cells within WAT are specific for the administered pathogen, suggesting that CD8^+^ T cells home to and persist (as CD8^+^ T_RM_ cells) within WAT after infection (193). Importantly, the authors also demonstrated that the induction of memory T cell responses in WAT results in the remodeling of WAT’s physiology in favor of the activation of antimicrobial response at the expense of lipid metabolism (193). These observations were the first to identify WAT as immune reservoirs sustaining pool of memory T cells and facilitating their reactivation, thus potentially contributing to immunological memory after infection. Whether WAT’s T_RM_ cells are important for recall responses to *T. gondii* and/or *Y. pseudotuberculosis* reinfections in humans remains to be seen.

Misumi and colleagues brought the second demonstration of an active anti-infectious role of ATs’ memory T cells (214). Following lymphocytic choriomeningitis virus infection, the authors showed that more memory IFNγ^+^ CD8^+^ T cells accumulate in the WAT of obese mice than in the WAT of lean mice. Strikingly, WAT memory T cells of obese mice rapidly caused lethal immunopathology upon re-challenge infection, whereas lean mice remained unaffected and could efficiently control the challenge (214). This demonstrates that obesity gives rise to an unusual form of T-cell-mediated pathogenesis during viral infection that leads to lethality.

Altogether, these major findings illustrate the importance of AT’s T cells to infection and in memory/recall responses and may have implications for the management of infections in individuals having alterations in AT’s immune cell composition and function, such as individuals with obesity and aged adults, who display impaired immune responses to pathogens.

In addition, unlike stereotypical lymphoid organs that primarily contain adaptive immune cells, ATs are enriched with several types of innate immune cells (e.g., macrophages, DCs), as well as with ILCs and innate-like T cells (e.g., iNKT cells, γδ T cells) (**Table 1**), which are likely contributing to host defense against infection. Indeed, adipose iNKT cells and γδ T cells have been reported to mediate host defense by modulating the number and/or function of other adipose immune cells (e.g., Tregs, DCs, NK cells and macrophages) (284), and DCs were shown to control adaptive immune responses to AT’s infection (133, 134, 285, 286).

## The special case of influenza A and SARS-CoV-2 respiratory viruses

Since the last twenty years, a growing number of new viruses has emerged and entered the human population (287). Moreover, influenza A (H1N1)pdm09 virus and severe acute respiratory syndrome coronavirus-2 (SARS-CoV-2) have caused global pandemics (288, 289). Epidemiological evidence establishes obesity and aging – two conditions associated with ATs dysfunctions – as important risk factors for increased susceptibility and severity to viral respiratory pneumonias associated with H1N1 and SARS-CoV-2 pandemics (290–292). This has prompted some researchers to (re)address the role of ATs in influenza and COVID-19. In this section, we will examine the impact of influenza virus and SARS-CoV-2 infection on ATs (**Table 2**), and comment on the contribution of ATs to influenza and COVID-19 pathogenesis.

Influenza A viruses (IAVs) are very contagious pathogens responsible for severe respiratory illnesses in humans and animals worldwide. IAVs are enveloped, segmented negative-sense, single-stranded RNA viruses that primarily infect respiratory epithelial cells (293). With the exception of the reports showing that IAV can infect adipocytes *in vitro* (272), and that IAV (H5N1) can target WAT *in vivo* (219), no study has investigated the impact of influenza infection on the WAT of lean mice until recently. Ayari and colleagues evaluated the metabolic consequences of IAV (H3N2) infection in lean mice, and the impact of infection on SCAT and VAT (220). The authors showed that IAV-infected mice present alterations in whole-body energy metabolism that persisted after the resolution of the infection. Importantly, during the acute phase of infection, viral RNA was detected in SCAT and, at a lower level, in VAT. Concomitantly, viral-antigen-harboring hematopoietic cells (i.e., expressing CD45, a leukocyte common antigen) were found in WAT (mostly in SCAT), and this was associated with the parallel activation of type I IFN signaling pathways and inhibition of cholesterol biosynthesis pathways, suggesting activation of antiviral innate immune responses. In addition, IAV infection induced a transient metabolic rewiring in SCAT (not VAT) – characterized by the emergence of UCP1-expressing thermogenic brown-like/beige adipocytes in the tissue. The latter may partly rely on a direct effect of the virus on SCAT: preadipocytes committed to the thermogenic differentiation program upon *in vitro* IAV infection (220). Since a growing body of evidence suggests that immune cells are highly sensitive to thermal stress (294, 295), SCAT browning may participate to the initial steps of immune defense against the infection. Thus, influenza-induced SCAT thermogenesis might help controlling antiviral immune defenses, locally. From the virus side, it is conceivable that, since the host expends energy to increase SCAT thermogenesis, it fails to support an efficient immune response against infection locally, thereby allowing the virus to propagate for a longer period of time in the tissue (296). At this stage, many questions remain unanswered about how the virus and/or infected cells reach the WAT, what is the phenotype of these cells, and do they participate to the host’s defense against infection?

In 2021, Zeng and colleagues reported that IAV can also infect the mesenteric VAT. In this fat pad, infection was associated with moderate decreased adipocyte number, increased release of leptin, visfatin and chemerin, and decreased release of adiponectin (221). The dissemination of IAV to ATs has been recently extended to the perivascular AT in a pregnant mouse model of infection (222). In this model, IAV preferentially disseminated into the perivascular AT of the abdominal aorta, and this was associated with the recruitment of proinflammatory monocytes and neutrophils, and the massive infiltration and activation of T cells. This suggests that during pregnancy, the perivascular AT might be a niche that supports IAV dissemination and a site of marked inflammation and vascular dysfunction, which may be central to the maternal and perinatal complications following IAV infection during pregnancy (297).

Altogether, these findings demonstrate that, during the acute phase of infection, IAV can disseminate to different adipose depots (SCAT and epidydimal VAT) (220), mesenteric VAT (221), and perivascular VAT of the abdominal aorta (222). To our knowledge, there is no published report on IAV targeting BAT.

Coronaviruses are responsible for mild to severe respiratory tract infections, similarly to influenza viruses (298). Coronaviruses are enveloped, non-segmented positive sense, single-stranded RNA viruses. To date, seven coronaviruses associated with human infections have been identified, such as the severe acute respiratory syndrome coronavirus-2 (SARS-CoV-2) (299), which caused the coronavirus disease 2019 (COVID-19) pandemic (300). Since adipose cells express ACE2 – the major cell entry receptor for SARS-CoV-2 (301) – and virus replication and its inflammatory insult are favored by the presence of lipid droplets (302), the hypothesis that ATs may serve as reservoir for storing and replicating the virus, as well as a site for cytokine amplification, has emerged as a potential explanation for the strong association between obesity, aging and COVID-19 severity (303, 304). Recent studies have shown that SARS-CoV-2 can indeed infect ATs’ cells, including adipocytes, thus favoring a local inflammatory response and resulting changes in lipid profiles (223–226, 305–307). In turn, these changes are thought to contribute to insulin resistance and to hamper patients’ recovery.

Reiterer and colleagues were the first to suggest that SARS-CoV-2 triggers WAT dysfunction, which in turn contributes to adverse COVID-19 outcomes (223). Indeed, these investigators reported that mouse (but not human) adipocytes infected with SARS-CoV-2 *in vitro* produced lower amounts of adiponectin – an insulin-sensitizing adipokine (308). Importantly, viral RNA and low adiponectin expression were evidenced in WAT from infected golden hamsters. These changes were associated with a robust inflammatory antiviral response in WAT and a systemic insulin-resistant state, suggesting that hyperglycemia in severe COVID-19 might result (at least in part) from infection-induced WAT dysfunction (223). Zickler and colleagues showed SARS-CoV-2 is frequently detectable in the WAT (SCAT and VAT) of patients deceased from COVID-19 (225). Remarkably, the virus was detected predominantly in the WAT of males who were overweight or obese. In female individuals, SARS-CoV-2 was also detected in WAT, although there was no clear correlation between fat mass and virus mRNA levels. In a hamster model of COVID-19, the authors also showed that SARS-CoV-2 spreads from the respiratory tract into WAT, where it continues to replicate, thereby leading to local inflammation and changes in whole-body metabolism. In *in vitro* infection experiments, the authors showed that ACE2 expression was strongly induced upon adipocyte differentiation, and provided mechanistic insight that lipid droplet metabolism is critical for SARS-CoV-2 propagation, since blocking lipid breakdown drastically reduced viral replication in mature adipocytes (225). Martinez-Colon and colleagues identified mature, lipid-laden adipocytes and macrophages as the two main cellular targets of SARS-CoV-2 in human WAT (226). Strikingly, preadipocytes are not permissive to infection – reinforcing the notion that lipid droplet metabolism is critical for SARS-CoV-2 propagation (225, 302). Colleluori and colleagues confirmed SARS-CoV-2’s ability to infect mature adipocytes and further showed that infected adipocytes are less viable and have a smaller lipid droplet size and a higher prevalence of pyknotic nuclei, which are suggestive of infection-induced cell delipidation and death (305). In human individuals who deceased from COVID-19, Basolo and colleagues recently confirmed the presence of the SARS-CoV-2 genome in the SCAT, and further showed that SARS-CoV-2 infection activates the IFN-alpha pathway and induces the recruitment of NK cells, macrophages and T cells in the tissue (306). Most recently, Saccon and colleagues confirmed that WAT (i.e., thoracic SCAT) is a frequent extrapulmonary site where SARS-CoV-2 can be detected in patients who died from COVID-19 (307). Using adipocytes differentiated from primary stromal-vascular cells isolated from the SCAT or the VAT of individuals undergoing bariatric surgery, the authors also provided evidence that VAT adipocytes are more susceptible than SCAT adipocytes to SARS-CoV-2 infection *in vitro (* 307). Although these observations were made *in vitro*, it appears that SARS-CoV-2 infection may have a depot-specific impact on ATs, likely due to the intrinsic differences between SCAT and VAT with regard to their cellular, molecular, and physiological characteristics, notably regarding lipid metabolism (47, 48 **)**. In a non-human primate model of COVID-19, SARS-CoV-2 was detected in SCAT but not in VAT or in epicardial AT (309). However, when evaluating the consequences of SARS-CoV-2 infection on WAT T cells, the authors showed that both SCAT and VAT T cells showed a drastic reduction in CD69 expression (309) – a standard marker of T cell activation and residency in tissues (310–312) that is expressed by most tissue resident memory T cells (312–314). Thus, the loss of CD69 expression on SCAT and VAT T cells in SARS-CoV-2-infected animals might reflect a major change in the migratory potential of adipose T cells, and/or functional alterations in adipose-resident-T cells caused by inappropriate activation (315). Besides, it is acknowledged that T cell activation requires a coordinated rewiring of cellular metabolism (to support increase in cell size and clonal expansion, lineage polarization, and acquisition of effector function) – a process that is likely to be dependent upon tissue’s metabolic rates and fuel requirements (316). Importantly, it has been reported that CD69 can function as a metabolic gatekeeper in T cells, notably regarding amino acid uptake (317). This demonstrates that in a model of mild infection, SCAT is selectively infected by SARS-CoV-2 although changes in the immune properties of AT are observed in both SCAT and VAT (309). Finally, while no reports are currently available on the detection of SARS-CoV-2 in BAT, it is noteworthy that ACE2 has been reported to be highly expressed in BAT, where it participates to the maintenance of thermogenesis and energy expenditure through the direct induction of UCP1 and activation of mitochondrial function (318).

Altogether, these findings demonstrated that ATs infection (mainly described for WAT) represents a relevant feature of COVID-19 and thus should be considered when investigating the mechanisms of disease pathogenesis (including immune responses), notably in at risk-populations such as obese and aged individuals.

## Concluding remarks

Historically viewed as inert sites for energy storage, adipose tissues (ATs) are now appreciated as important regulators of many aspects of whole-body physiology, including immune responses. In this review, we remind that ATs harbor a plethora of innate and adaptive immune cells that shape their endocrine and metabolic functions and contribute to tissue repair and homeostasis as well as to immunosurveillance and immunoregulation. These last years, a wide range of pathogens (bacteria, parasites or viruses such as the respiratory viruses influenza and SARS-CoV-2) have been reported to target ATs, and eventually persist within. Notably, since ATs express ACE2 and SARS-CoV-2 replication is favored by the presence of lipid droplets, the hypothesis that ATs may constitute major reservoirs for viral shedding/spread and potentiators of local and systemic inflammation, has emerged as a possible explanation for the severe forms of COVID-19 in obese and aged individuals as well as for the enduring post-infection symptoms collectively termed long COVID. More generally, the demonstration that both some infections can persist in ATs that act as sources of subsequent disease, expands the knowledge of host-pathogen interactions, in which ATs should not be ignored. Beside inducing metabolic and inflammatory changes in the tissue, we describe how these infections also lead to alterations in the adipose immune landscape. We finally touch upon recent groundbreaking evidence implicating adipose resident memory T cells in host defense against certain pathogens, which show how vital the adipose immune system is for host defense against infection. The translatability of these major breakthroughs to humans still remains to be established. Nevertheless, these fascinating results might allow to understand the differences in disease pathogenesis in fragile populations such as the elderly and obese individuals – who present with quantitative and qualitative changes in adipose immune cells. Increasing our understanding of the role of ATs immune cells might ultimately lead to the development of innovative preventive/therapeutic strategies (targeted immunomodulation) for the treatment of infectious diseases and effective vaccination in at-risk populations.

## Author contributions

All authors listed have made a substantial, direct, and intellectual contribution to the work (reviewing of literature, writing and editing the manuscript, designing Figures and Tables), and approved the submitted version of the manuscript.
